# Characterization of the bacterial microbiome of non-hematophagous bats and associated ectoparasites from Brazil

**DOI:** 10.3389/fmicb.2023.1261156

**Published:** 2023-10-19

**Authors:** Marcos Rogério André, Priscila Ikeda, Daniel Antônio Braga Lee, Renan Bressianini do Amaral, Lucas Amoroso Lopes Carvalho, Daniel Guariz Pinheiro, Jaire Marinho Torres, Victória Valente Califre de Mello, Gregory K. Rice, Regina Z. Cer, Elizabete Captivo Lourenço, Carisa Elisei Oliveira, Heitor Miraglia Herrera, Darci Moraes Barros-Battesti, Rosangela Zacarias Machado, Kimberly A. Bishop-Lilly, Clifton L. Dalgard, J. Stephen Dumler

**Affiliations:** ^1^Vector-Borne Bioagents Laboratory (VBBL), Departamento de Patologia, Reprodução e Saúde Única, Faculdade de Ciências Agrárias e Veterinárias (FCAV), Universidade Estadual Paulista (UNESP), Jaboticabal, São Paulo, Brazil; ^2^Departamento de Biotecnologia Ambiental e Agropecuária, Faculdade de Ciências Agrárias e Veterinárias (FCAV), Universidade Estadual Paulista (UNESP), Jaboticabal, São Paulo, Brazil; ^3^Laboratório de Biologia Parasitária, Programa de Pós-Graduação em Biotecnologia, Universidade Católica Dom Bosco, Campo Grande, Mato Grosso do Sul, Brazil; ^4^Leidos, Inc., Reston, VA, United States; ^5^Department of Genomics and Bioinformatics, Naval Medical Research Command, Fort Detrick, Frederick, MD, United States; ^6^Laboratório de Ecologia de Mamíferos, Universidade do Estado do Rio de Janeiro, Rio de Janeiro, Brazil; ^7^The American Genome Center, Center for Military Precision Health and Department of Anatomy, Physiology, and Genetics, Uniformed Services University of the Health Sciences, Bethesda, MD, United States; ^8^Department of Pathology, University of the Health Sciences, Bethesda, MD, United States

**Keywords:** microbiome composition, Chiroptera, bat flies, mites, Macronyssidae, Spinturnicidae, Streblidae

## Abstract

**Introduction:**

Bats, along with their ectoparasites, harbor a wide diversity of symbiotic and potential pathogenic bacteria. Despite the enormous diversity of bats (181 species), few studies aimed to investigate the bacterial microbiome of Brazilian chiropterans and associated ectoparasites. This study aimed to characterize the bacterial microbiome of non-hematophagous bats and associated Streblidae flies and Macronyssidae and Spinturnicidae mites in the state of Mato Grosso do Sul, midwestern Brazil.

**Methods:**

Oral and rectal swabs were collected from 30 bats (*Artibeus lituratus* [*n* = 13], *Artibeus planirostris* [*n*  =  9], *Eptesicus furinalis* [*n* = 5], *Carollia perspicillata* [*n* = 2], and *Platyrrhinus lineatus* [*n* = 1]). In addition, a total of 58 mites (15 Macronyssidae and 43 Spinturnicidae) and 48 Streblidae bat flies were collected from the captured bats. After DNA extraction and purification, each sample’s bacterial composition was analyzed with metagenomic sequencing.

**Results:**

The microbiome composition of both oral and rectal bat swab samples showed that Gammaproteobacteria was the most abundant bacterial class. *Spiroplasma, Wolbachia* and *Bartonella* represented the most abundant genera in Streblidae flies. While *Wolbachia* (Alphaproteobacteria) was the most abundant genus found in Spinturnicidae, *Arsenophonus* (Gammaproteobacteria) was found in high abundance in Macronyssidae mites. In addition to characterizing the microbiome of each sample at the class and genus taxonomic levels, we identified medically significant bacteria able to infect both animals and humans in oral (*Streptococcus* and *Anaplasma*) and rectal swabs (*Enterobacter*, *Klebsiella*, *Escherichia*, *Enterococcus*, *Streptococcus*), Macronyssidae (*Anaplasma*, *Bartonella*, *Ehrlichia*) and Spinturnicidae (*Anaplasma*, *Bartonella*) mites as well as Streblidae flies (*Spiroplasma*, *Bartonella*).

**Discussion and conclusion:**

Besides expanding the knowledge on the bacterial microbiome of non-hematophagous bats and Streblidae flies from Brazil, the present work showed, for the first time, the bacterial community of bat-associated Macronyssidae and Spinturnicidae mites.

## 1. Introduction

Bats (Mammalia, Chiroptera) play an important ecological role in many different ecosystems, especially as pollinators and seed dispersers (Ramírez-Fráncel et al., 2022), as well for their importance as potential vectors and natural reservoirs for several pathogens (e.g., virus, bacteria, fungi) (Han et al., 2015; Allocati et al., 2016) and hosts for ectoparasites (e.g., flies, ticks, mites) (Stuckey et al., 2017). In addition to their large number of species (1,460 worldwide; 181 in Brazil) (Garbino et al., 2022; Upham et al., 2023) and broad geographic distribution, bats can also live in various habitats (wild, rural and urban) and have different dietary strategies (insectivore, carnivore, omnivore, frugivore, nectarivore, piscivore and hematophagy), which favor the exposure to a wide variety of environments and microorganisms (Avena et al., 2016; Alcantara et al., 2022).

Just like other mammals, many biological functions, such as immune system efficiency (Zhang et al., 2013; Thaiss et al., 2016), digestion, and individual development and growth, are influenced by the coevolution of these animals with microorganisms, especially bacteria (Lutz et al., 2022). For example, bats have been implicated as the likely ancestral hosts of all *Bartonella* bacteria associated with mammals and seem to have played a significant role in the initial geographic spread of this genus. Their coevolution with *Bartonella* might have had a profound impact on the bacteria evolutionary diversification and their transmission to other orders of mammals, including humans (McKee et al., 2021).

Since bats live in association with many different bacteria, the composition of their microbiomes is directly influenced by environmental (e.g., geographic location, climatic conditions) (Lemieux-Labonté et al., 2016; Lutz et al., 2022), behavioral (e.g., diet, frequented habitats) (Carrillo-Araujo et al., 2015; Corduneanu et al., 2023) and individual (e.g., species, age) features (Hughes et al., 2018; Jones et al., 2022).

While the microbiome’s significance in bat adaptation, survival and evolution is undeniable, many bat-associated microorganisms can be pathogenic to other animals, including humans (Dimkić et al., 2021). Among pathogenic agents, the Phylum Proteobacteria stands out and comprises Gram-negative bacteria with several different genera of phototrophic, plant- and arthropod-associated symbionts, as well as pathogens. This phylum is sub-divided into six different classes and comprises important pathogenic/zoonotic genera commonly detected in bats, such as *Anaplasma*, *Ehrlichia*, *Neorickettsia* (Alphaproteobacteria, Anaplasmataceae), *Bartonella* (Alphaproteobacteria, Bartonellaceae) and *Coxiella* (Gammaproteobacteria, Coxiellaceae) (Cicuttin et al., 2013; Rizzatti et al., 2017; Ferreira et al., 2018; Ikeda et al., 2020, 2021).

The microbiome composition of many biological samples (feces, skin, urine, organs, fluids) collected from over 200 bat species (mostly insectivorous bats) from 32 different countries have already been assessed (Jones et al., 2022). Many authors report a high abundance of Proteobacteria in bat gut flora. In South Africa, Mehl et al. (2021) analyzed the composition of intestinal bacteria of insectivorous bats (*Neronomica nana*) sampled on wastewater treatment work and reference sites. The authors reported Proteobacteria abundance (range of 19.9 to 46.6%) comprising four different classes (Alphaproteobacteria, Betaproteobacteria, Epsilonproteobacteria and Gammaproteobacteria). Additionally, the authors reported that the bat sampling site was the most influential parameter on microbiome constitution and diversity (Mehl et al., 2021).

The gut microbiome composition of two insectivorous bat species (*Rhinolophus sinicus* and *Myotis altarium*) was described and compared according to different gut sections (i.e., small and large intestine) and feces. Proteobacteria was the dominant phylum in both bat species (average of 43.5% in *R. sinicus* and 42.5% in *M. altarium*), comprising over half of the microbiome composition in the large intestine of *M. altarium* and feces of *R. sinicus* (Wu et al., 2019). Indeed, Hughes et al. (2018) demonstrated the high presence of Proteobacteria (42.8–57.4%) in bat guts in comparison to other mammals, when analyzing rectal swab samples from *Myotis myotis* insectivore bats from western France.

Although the majority of studies on bat-associated microbiomes have been performed on gut-associated samples, such as guano or small and large intestines, it is crucial to elucidate the microorganism composition of other body sites aiming at gaining a better understanding of coevolution, ecology and pathogen transmission (Lutz et al., 2019; Dimkić et al., 2021). For instance, potentially zoonotic bacteria were discovered in saliva, urine and feces of insectivorous bats from South Africa (Dietrich et al., 2017). Proteobacteria were detected in all examined body sites with different sequence abundances (>90% in saliva; 32.6% in urine; 31.2% in feces), including some in clades with established zoonotic arthropod-borne bacteria, such as *Bartonella*, *Rickettsia,* and *Coxiella* (Dietrich et al., 2017).

The bat skin microbiome is also inhabited by a high abundance of Proteobacteria. Winter et al. (2017) used whole body cotton swabs from cave bats in the southwestern United States to evaluate skin and fur microbiome. Proteobacteria comprised 24.6% of the microbiota, ranging from 8.15 to 39.98%, with Alphaproteobacteria representing the most abundant class. A higher abundance of Proteobacteria in the skin microbiome of bats (> 60%), using whole body cotton swabs, was also reported in the Northeastern region of the United States (Avena et al., 2016).

Proteobacteria showed abundance ranging from 1.31 to 99.7% among 10 heart samples (8 individual and 2 pooled) of insectivorous bats from Central and South Eastern Europe (Corduneanu et al., 2021). Furthermore, specific PCR assays based on the *gltA* and *rpoB* encoding genes and ITS region proved the presence of *Bartonella* sp. in one pooled heart sample; additionally, PCR assays targeting the *gltA* and *ompA* genes confirmed the presence of *Rickettsia* sp. in one individual sample.

In Brazil, studies on the composition of bat-associated microbiomes are scarce. Cláudio et al. (2018) described the oral and rectal cavity-associated microbiota from bats sampled in southeastern Brazil, which were divided into five feeding guilds (insectivores, frugivores, nectarivores, carnivores, and hematophagous). After isolating bacteria, taxon identification was performed by matrix-assisted laser desorption/ionization (MALDI-TOF) technique. Proteobacteria represented over 87% of the microbiome from bats presenting all dietary strategies, without further identification of the bacterial classes/genera. Additionally, previous studies in Brazil have already detected, by using PCR assays followed by sequencing and phylogenetic inferences, potential zoonotic Proteobacteria in Brazilian bats, namely *Bartonella* spp. (Ikeda et al., 2017, 2020; André et al., 2019), *Ehrlichia* sp. (Ikeda et al., 2021), *Neorickettsia* sp. (Ikeda et al., 2021), *Anaplasma* spp. phylogenetically associated to *A. phagocytophilum* and *A. bovis* (Ikeda et al., 2021), and *Coxiella burnetti* (Ferreira et al., 2018).

Moreover, bats are hosts for many hematophagous ectoparasites, including flies (Diptera: Streblidae and Nycteribiidae), soft ticks (Ixodida: Argasidae) and mites (Mesostigmata: Macronyssidae and Spinturnicidae), whose microbiome composition is also of great significance (Szentiványi et al., 2019). Although the diversity of bacteria that comprise those microbiomes is mainly associated with the evolution and ecology of ectoparasite species, recent studies suggest that this composition is also influenced by environmental factors and host ecology (Speer et al., 2022). The investigation of the microbiome composition of seven different Nycteribiidae bat fly species in the Malagasy Region revealed that Alphaproteobacteria represented 17% of the identified species, while Betaproteobacteria and Gammaproteobacteria constituted 3 and 78%, respectively, with 55% of the sequences corresponding to *Wolbachia*, 26% to *Bartonella*, and 17% to *Rickettsia*. Phylogenetic analyses, based on the concatenation of *fbpA*, *ftsZ* and *hcpA* genes, positioned *Wolbachia* sequences into the F subgroup, which comprises strains associated with many different arthropod orders (Diptera, Scorpiones, Hemiptera, Coleoptera, Phthiraptera). The *gltA*-based phylogeny clustered the *Bartonella* genotypes into five different groups along with similar bat and bat-fly associated sequences (Wilkinson et al., 2016).

In Brazil, Speer et al. (2022) reported a significant difference in the bacterial microbiome composition of Streblidae and Nycteribiidae flies collected from bats in the Atlantic Forest. Based on next generation sequencing (NGS) of the V4 region of the 16S rRNA gene, Alphaproteobacteria were highly abundant in nycteribiid bat flies, mainly *Wolbachia* and *Bartonella* (> 75%). Both genera were also detected in streblid bat flies, but at a much lower prevalence (> 10%). *Arsenophonus* (Gammaproteobacteria) showed a higher abundance in streblids (> 70%) when compared to nycteribiid flies (> 1%).

Although the microbiome composition of bat mites has not yet been studied, these arthropods are known to act as hosts for many pathogenic agents. Two studies carried out in China demonstrated that Spinturnicidae bat mites (*Spinturnix* sp. and *Eyndhovenia* sp.) collected from insectivorous bats harbored *Bartonella* genotypes associated with *Bartonella mayotimonensis* (Han et al., 2022) as well as hemoplasma genotypes, including one with high similarity to the human-pathogenic ‘*Candidatus Mycoplasma haemohominis*’ (Wang et al., 2023). In midwestern Brazil, Ikeda et al. (2020) demonstrated the presence of *Ehrlichia* sp., closely related to *E. ruminantum* and *E. minasensis*, in Macronyssidae (*Steatonyssus* sp.) and Spinturnicidae (*Periglischrus* sp., *Periglischrus torrealbai* and *Periglischrus acustisternus*) mites collected from non-hematophagous bats and analyzed using PCR assays based on the *dsb* gene.

Considering the role of bats as carriers of pathogens with medical and veterinary importance (Federici et al., 2002; Van Brussels and Holmes, 2002), the assessment of the bacterial microbiome composition of bats and associated ectoparasites can contribute to the understanding of the mechanisms involved in the adaptability and coevolution of this group of mammals with symbionts and pathogenic agents. Therefore, the present work aimed to investigate the bacterial microbiome composition of non-hematophagous bats and associated ectoparasites sampled in midwestern Brazil.

## 2. Materials and methods

### 2.1. Bats sampling: swabs and ectoparasites

**Figure 1:**
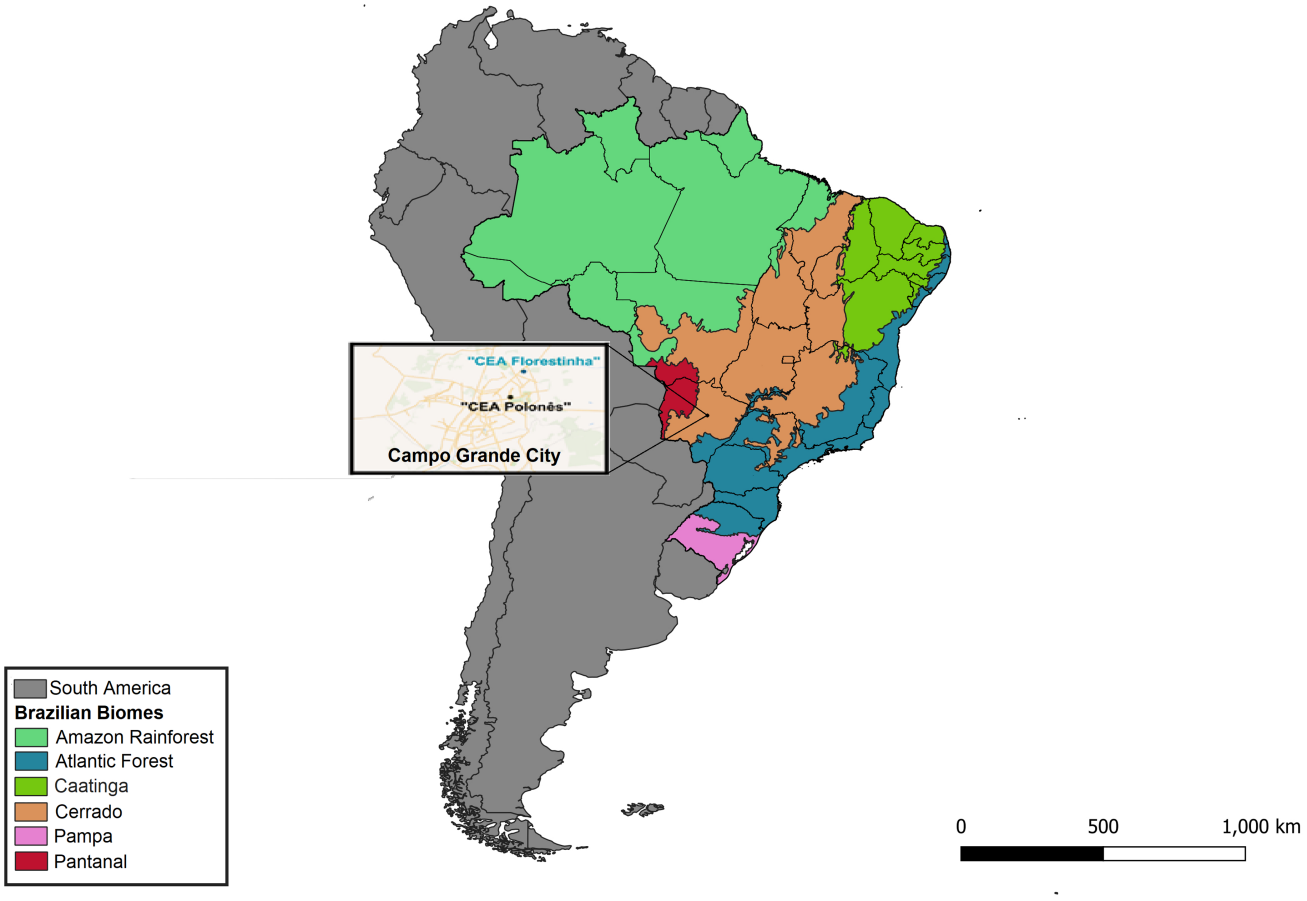
Map of the collection locations (“CEA Florestinha” and “Polonês”) for biological samples and ectoparasites from non-hematophagous bats in the city of Campo Grande, Mato Grosso do Sul state, Brazil.

Ectoparasites (*n* = 106) were sampled from 82 out of 135 bats captured between June, 2017 and March, 2018 in two periurban regions of Campo Grande city, Mato Grosso do Sul state, central-western Brazil, namely “Centro de Educação Ambiental Polonês” (54°34′49.816”W and 20°26′50.673”S) and “Florestinha” (54°33′43.352”W and 20°24′11.614”S) (Figure 1), under authorization of ICMBio – SISBIO number 57450–1 and Ethics Committee on the Use of Animals (CEUA – FCAV / UNESP), registered under number 010050. The captures were performed using seven mistnets (Zootech©), with 3×12 m size and 20 mm mesh, per night, as described by Kunz and Kurta (1988) and Peracchi and Nogueira (2010). Once opened, nets were checked every ~30 min for six hours of sampling effort per night. The captured bats were carefully restrained and examined in field where all the ectoparasites were collected using tweezers and stored in RNAse- and DNAse-free microtubes (Kasvi©) containing absolute alcohol (Merck©). Bats and associated ectoparasites were identified using identification keys (Herrin and Tipton, 1975; Wenzel, 1976; Guimarães, 2001; Reis et al., 2017).

Oral and rectal swab samples were collected only from bats (*n* = 30) captured in March, 2018. Swabs were collected in a laminar flow hood, using sterile swabs and stored in RNAse- and DNAse-free microtubes (Kasvi©) containing RNA Stabilization Solution (Invitrogen™, Thermo Scientific©) and kept at −80°C. All samples were collected in a laminar flow biosafety cabinet, using appropriately sterilized instruments to avoid environmental contamination.

### 2.2. DNA and RNA purification

The ectoparasites were used only to prepare DNA using the Illustra Tissue and Cells Genomic Prep Mini Spin Kit (GE Healthcare©), following the manufacturer’s recommendations.

To allow for not only the bacterial component of the bat metagenome to be analyzed in the current study, but also for potential future analysis of viral components, oral and rectal swabs were used to prepare total nucleic acids (DNA/RNA; tNA) with the ZymoBIOMICS DNA/RNA Miniprep Kit (ZymoResearch©), according to the manufacturer’s recommendations. The tNA samples were then used for cDNA synthesis using the Invitrogen SuperScript ™ III kit and random hexamers (Invitrogen ™, Thermo Scientific©) for the first strand and NEBNext® Ultra™ II Non-Directional RNA Second Strand Synthesis Module (New England BioLabs©) for the second strand. The nucleic acid samples were purified using AMPure XP beads (Beckman Coulter Life Sciences©). Prior to library preparation, the concentration of all nucleic acid samples was assessed by dsDNA Qubit™ Assay (Invitrogen ™, Thermo Scientific©).

### 2.3. Library preparation and sequencing

Nucleic acid samples were used for library preparation and metagenomic sequencing using tagmentation that was performed according to the manufacturer’s recommendations (Illumina© DNA Prep Kit, Illumina©). Each library was dual indexed using the IDT® for Illumina® DNA/RNA UD Indexes Set A and Set B (Illumina©).

After library preparation, the samples were submitted for Fragmentation Analysis to assess the quality of each library. This step was performed using the Fragment Analyzer using the NGS Fragment Kit (1-6000 bp) (Agilent Technologies©), aiming to quantitatively and qualitatively analyze NGS libraries from 100 to 6,000 bp. The libraries that showed good fragment analysis quality, with one single peak and demonstrating that the library has homogenous size, were submitted for quantitative PCR with posterior pooling in accordance with each quantity and molecular weight. Sequencing was performed using Illumina NextSeq technology with 2x150bp to target roughly 4 M reads per sample.

### 2.4. Metagenome assembly and taxonomic assignment

Initially, the raw sequences had their qualities and quantities assessed with FastQC (v.0.11.5; Andrews et al., 2015). Pre-processing removed sequencing adapters with the program Atropos (v.1.1.21; Didion et al., 2017), using the modes “insert” and “adapter” sequentially. Next, Prinseq-lite (v.0.20.4; Schmieder and Edwards, 2011) was used for pruning and removing low-quality sequences. For metagenomic assembly, pre-processed libraries were grouped according to sample source: bat swabs (oral and fecal) and ectoparasite groups (flies, Macronyssidae mites and Spinturnicidae mites). The metagenomes were assembled with MEGAHIT (v.1.2.2-beta; Li et al., 2016) using default options and the “--presets meta-sensitive” parameter. Additionally, the “fusion” module of the SOAPdenovo2 (v.2.04; Luo et al., 2015) was used to perform the scaffolding of the assembled contigs, with a “K” value of 41. The reads were aligned against the reference metagenomes using the BWA-MEM algorithm (v.0.7.15; Li, 2013).

The quality and completeness of metagenomic assemblies were assessed using the MetaQUAST tool (v5.0.2; Mikheenko et al., 2016). The execution followed the default parameters established by the program’s authors, except for including the “--fragmented” flag, as the reference assembly was based on contigs and scaffolds. MetaQUAST generated a comprehensive report, which included quality metrics such as the number of contigs, N50, L50, and the alignment of reads to the metagenomic assemblies.

For abundance quantifications, we initially generated an index using the “index” function of the Kallisto program (v0.46.2; Bray et al., 2016). Afterward, we employed the “quant” function of the same program to quantify abundance based on normalized counts of contigs and scaffolds. In addition to the required parameters (index [−-index], output directory [−-output-dir], and input sequences), we included the bootstrap step [−-bootstrap-samples 100] to enhance the robustness of abundance estimates. The remaining parameters followed the default values established by the authors.

The sequences were submitted for taxonomic assignment using Kraken2 (v.2.0.8-beta; Wood et al., 2019) against NCBI reference databases (all reference genomes available until March 2022, with a status of “complete genome” or “chromosomes”). With the count tables and taxonomies, we performed data wrangling with a custom R script using the package “tidyverse” (v.1.3.1; Wickham et al., 2019) to make the outputs compatible with the MicrobiomeAnalyst platform (Dhariwal et al., 2017), for microbiome analyses. The scripts and parameters used are available in the repository: https://github.com/bioinfo-fcav/bat-microbiome.

## 3. Results

### 3.1. Bat biological samples and ectoparasites

A total of 106 ectoparasites were collected from 82/135 (60.74%) animals during fieldwork. For the present work, DNA samples from 58 pooled mites (*Periglischrus iheringi* [*n* = 29], *Steatonyssus* sp. [*n* = 15], *Periglischrus* sp. [*n* = 5], *Periglischrus torrealbai* [*n* = 5], *Periglischrus acutisternus* [*n* = 4]) (Supplementary Table S1), and 48 bat flies (*Megistopoda aranea* [*n* = 18], *Trichobius costalimai* [*n* = 14], *Trichobius dugesii complex* [*n* = 9], *Trichobius parasiticus complex* [*n* = 2], *Trichobius joblingi* [*n* = 2], *Strebla hertigi* [*n* = 2], *Paratrichobius longicrus* [*n* = 1]) (Supplementary Table S2) were used.

Oral and rectal swab samples were collected from 30 animals (*Artibeus lituratus* [*n* = 13], *Artibeus planirostris* [*n* = 9], *Eptesicus furinalis* [*n* = 5], *Carollia perspicillata* [*n* = 2], and *Platyrrhinus lineatus* [*n* = 1]) captured in the last fieldwork, totaling 60 samples (two from each animal). As bats are increasingly recognized as reservoirs of RNA viruses, total nucleic acid was extracted from these samples to allow for future viral analyses. Post tNA extraction, four rectal samples were not suitable for proceeding with library preparation based on nucleic acid quality control results and were therefore excluded.

In total, 162 libraries were prepared from the DNA samples obtained from ectoparasites (*n* = 106; 58 mites and 48 bat flies) and from the tNA oral and fecal swabs samples (*n* = 56; 30 oral swabs and 26 fecal swabs).

### 3.2. Sequence processing, quality control, and metagenome assembly

In this study, high-throughput sequencing of 168 samples from bats and their ectoparasites generated 245.4 Gbp of data. The samples yielded an average of 4,868,999 raw read pairs. After quality control, an average of 4,710,016 high quality read pairs were considered for further analysis. These processed and filtered reads were used to assemble the metagenomes, which were grouped according to their origin. The metagenome assembly process resulted in 2,684,052 contigs larger than 1 Kbp. The processing and assembly information for each metagenome is presented in Supplementary Table S3.

### 3.3. Rarefaction curve and statistical analyses

The absence of a plateau phase in the rarefaction curves suggests inadequate sequencing depth, which made statistical comparisons of taxon percentages unreliable.

### 3.4. Oral and fecal microbiome samples

**Figure 2:**
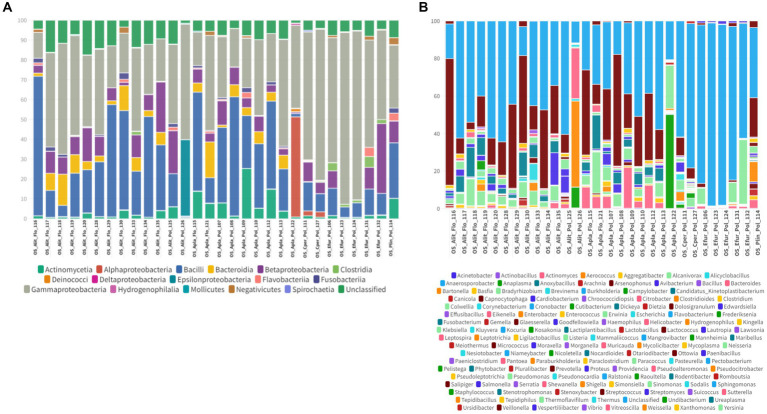
**(A)** Percentage of all taxa at the class level found in the different oral swab samples from bats. **(B)** Percentage of all taxa at the genus level found in the different oral swab samples from bats.

**Figure 3:**
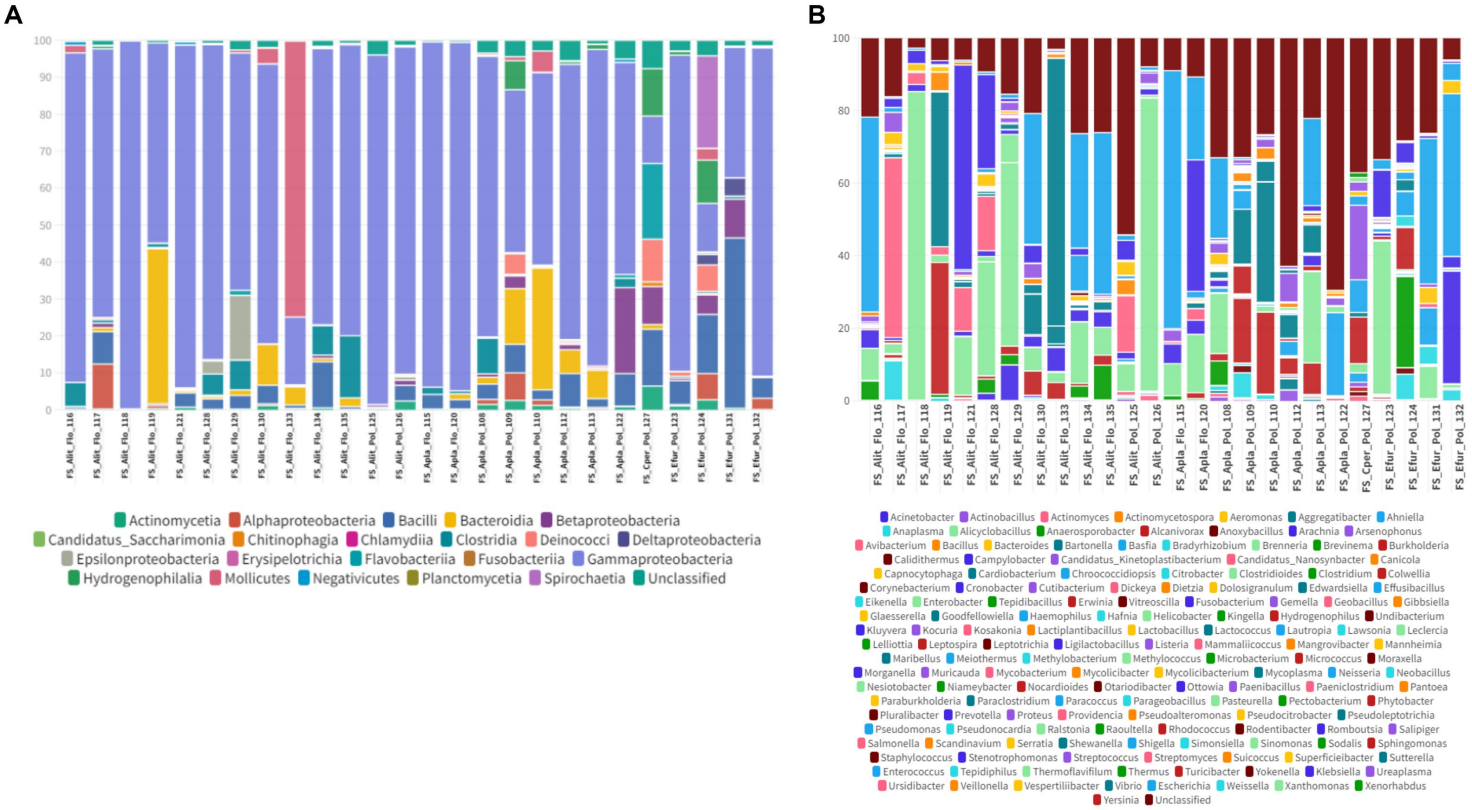
**(A)** Percentage of all taxa at the class level found in the different fecal swab samples from bats. **(B)** Percentage of all taxa at the genus level found in the different fecal swab samples from bats.

In the oral swab samples, 18 taxa were identified at the class level and 157 taxa at the genus level (Figures 2A,B). In the fecal swab samples, a total of 21 taxa were found at the class level and 189 at the genus level (Figures 3A,B).

**Figure 4:**
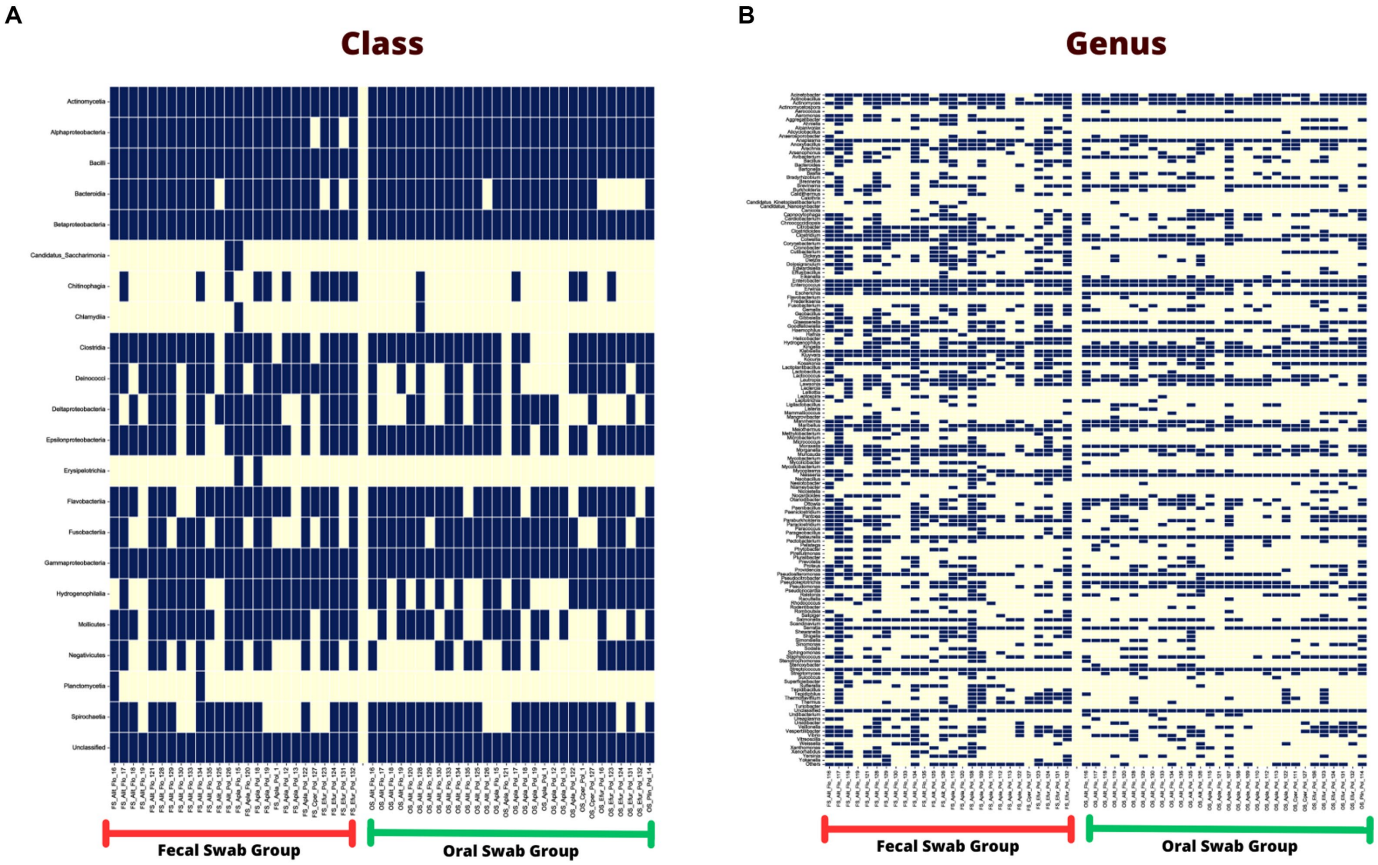
**(A)** Occurrence map of each taxon per sample at the class level found in the fecal and oral swab samples from bats. **(B)** Occurrence map of taxa at the genus level found in the fecal and oral swab samples from bats.

Among the classes found in the oral swabs, all were also present in the fecal swabs. However, the classes ‘*Candidatus* Saccharimonia’, Erysipelothia, and Planctomycetia were only found with at least one occurrence in some fecal swab samples, but not in the oral swabs (Figure 4A).

At the genus level, 145 taxa were shared, with at least one occurrence, between the fecal and oral swab samples, whereas 45 genera were detected exclusively in fecal swabs and 12 taxa were exclusively present in oral samples (Figure 4B).

Furthermore, in the 30 oral swab samples, the occurrence of five taxa at the class level was observed as following: Gammaproteobacteria with an average abundance of 42% (range 13–86%), Bacilli with 26% (0.5–70%), Betaproteobacteria with 8% (0.02–35%), Actinomycetia with an average of 4% (0.2–25%), Alphaproteobacteria with 1% (0.02–50%), and unclassified sequences with an average of 8% (1.4–17.5%) (Figures 2A, 4A). For the 26 fecal swab samples, four taxa at the class level were identified: Gammaproteobacteria with an average abundance of 70% (13–99%), followed by Bacilli with 6% (0.01–46%), Actinomycetia with 4% (0.005–6%), and Betaproteobacteria with 2.3%. Additionally, an average of 2.2% (0.1–8%) of unclassified taxa was found (Figures 3A, 4A).

Upon taxonomic classification of sequences found in the oral swab samples at the genus level, 49% (11–97.5%) of the taxa were unclassified. Among the genera found in the 30 oral swab samples, the following stand out: *Streptococcus* with an average abundance of 19.7% (0.07–67%), *Neisseria* with 3% (0.003–34.3%), *Enterobacter* with 1.9% (0.0042–45.8%), and *Anaplasma* with 1.45% (0.02–49.9%). In 29 out of 30 oral swab samples, *Actinomyces* showed an average abundance of 2.8% (0.004–22.8%), *Klebsiella* with 1.4% (0.005–22.8%), *Kluyvera* with 0.5% (0.005–8%), and *Pasteurella* with 0.1% (0.01–0.4%) (Figures 2B, 4B).

Similar to the oral swab samples, the fecal swab samples also exhibited a high percentage of unclassified taxa at the genus level, with an average of 23.6%. The most prominent taxa in terms of abundance per sample and percentage of detections in the 26 fecal swab samples were *Enterobacter*, with an average of 21% (0.002–85%), and *Streptococcus* with 1.6% (0.001–5.4%). Additionally, it was possible to detect the occurrence of *Klebsiella* in 25 out of the 26 fecal swab samples, with an average of 6% (0.003–56%), and *Serratia* with 1.3% (<0.001–4.3%). In 24 out of 26 samples, *Enterococcus* was detected with 2.5% (0.03–40%) and *Escherichia* with 1.3% (0.01–53%). In 23 out of 26 samples, *Actinomyces* with 0.2% (<0.001–1.2%) and *Anaplasma* with 1.2% (<0.01–7%) (Figures 3B, 4B).

### 3.5. Ectoparasite samples

#### 3.5.1. Mites

From the microbiome analysis we detected 20 bacterial taxa and 182 genera in Macronyssidae mites and 9 classes and 52 species from Spinturnicidae mites. All the classes and genera detected in spinturnicids were also present in the microbiome composition of macronissids, except for the genera *Actinotignum*, *Aeromonas*, *Alcaligenes*, *Citrobacter*, *Fenollaria* and *Sphingorhabdus*, which were exclusively found in Spinturnicidae.

**Figure 5:**
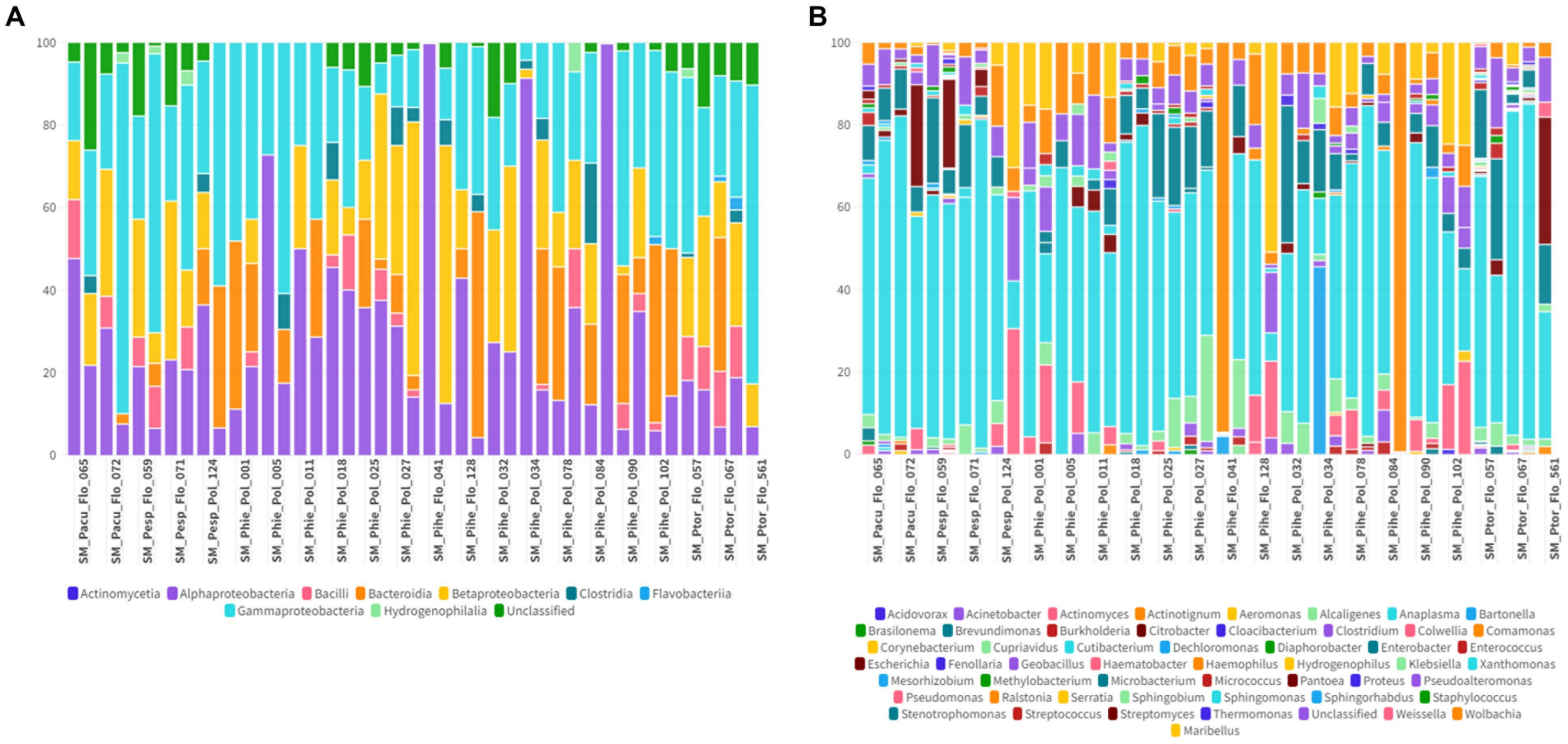
**(A)** Percentage of all taxa at the class level found in Spinturnicidae mites collected from bats. **(B)** Percentage of all taxa at the genus level found in Spinturnicidae mites collected from bats.

**Figure 6:**
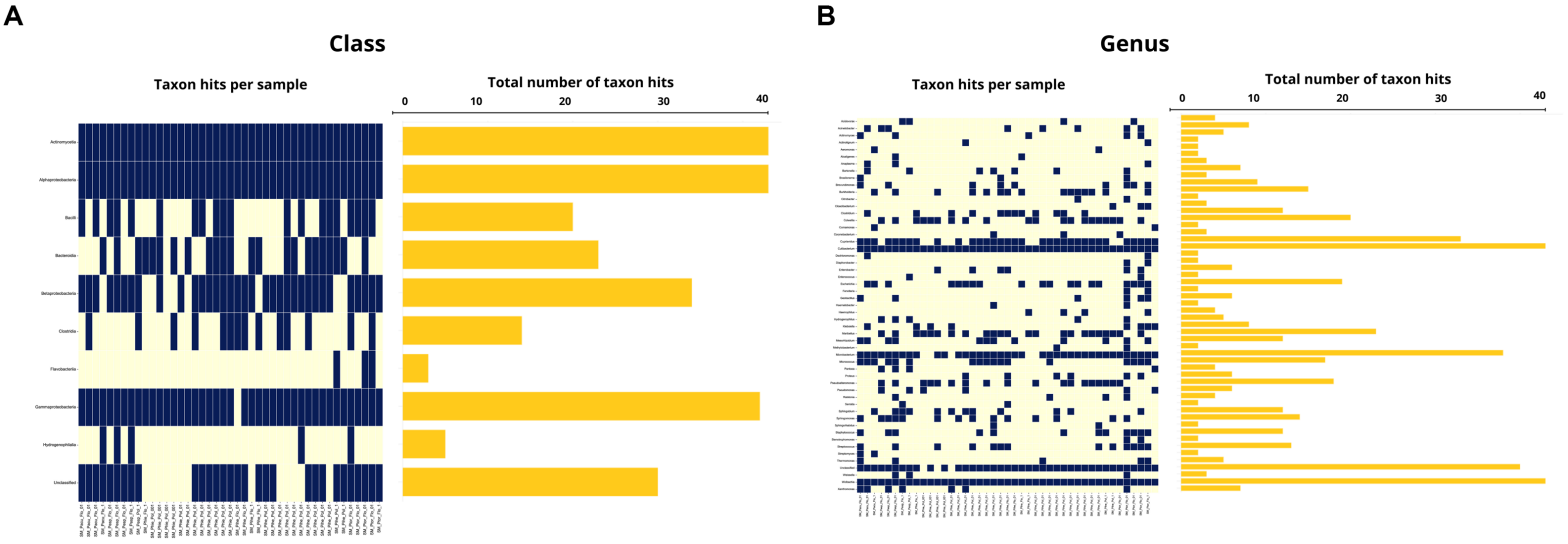
**(A)** Occurrence map of each taxon per sample at the class level found in the Spinturnicidae mite samples. **(B)** Occurrence map of taxa at the genus level found in the Spinturnicidae mite samples.

Among the classes detected in the microbiome of spinturnicids the following stand out: Alphaproteobacteria with an average abundance of 67.2% (1.7–99.4%), Actinomycetia with an average abundance of 24.2% (0.2–89.4%) and Gammaproteobacteria with an average abundance of 3.7% (0–52.2%) (Figures 5A, 6A). Regarding the most abundant genera, *Wolbachia*, *Cutibacterium* and *Microbacterium* showed average abundances of 65.3% (0.4–94.7%), 20.3% (0.1–80.3%) and 3.15% (0–20.7%), respectively (Figures 5B, 6B).

**Figure 7:**
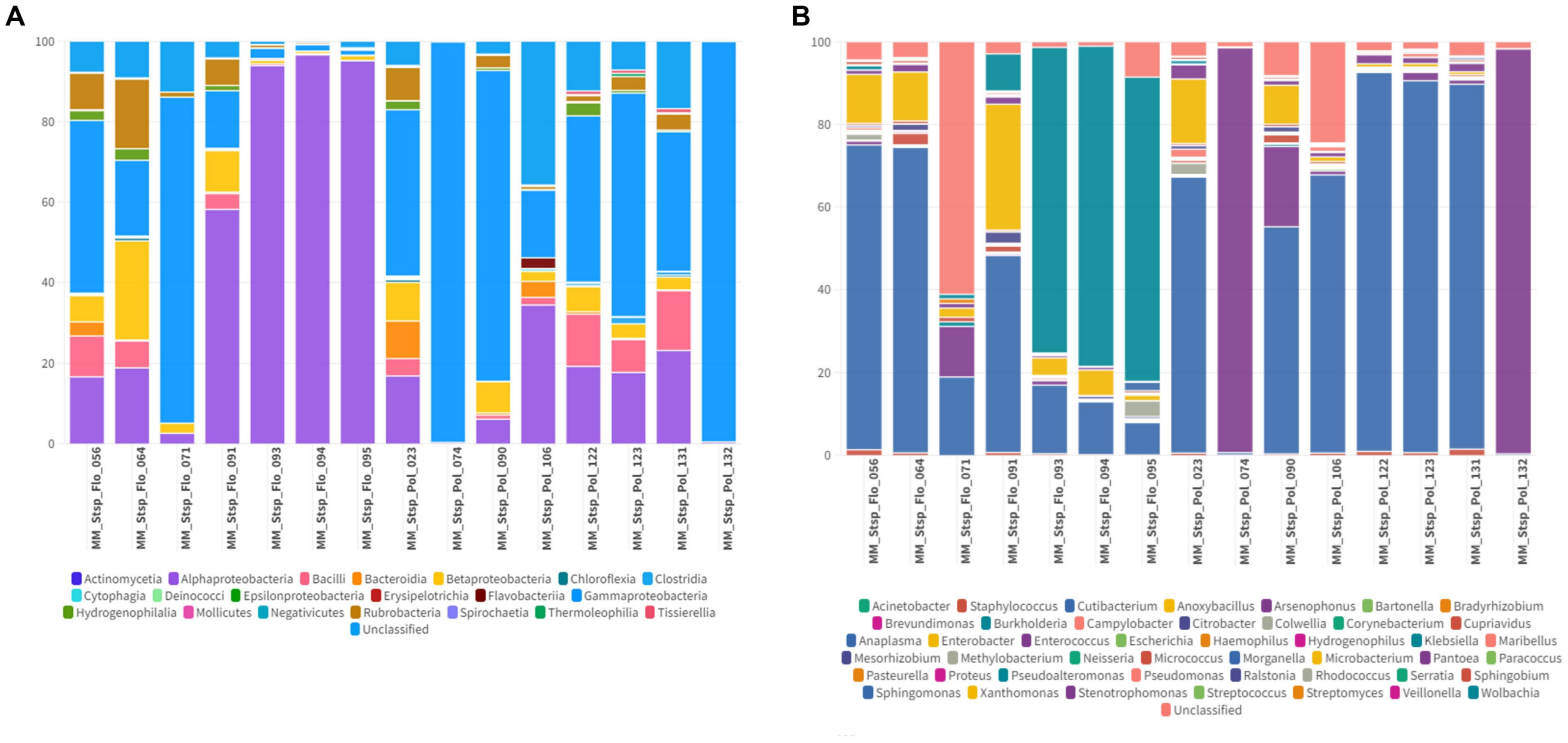
**(A)** Percentage of all taxa at the class level found in Macronyssidae mites collected from bats. **(B)** Percentage of all taxa at the genus level found in Macronyssidae mites collected from bats.

**Figure 8:**
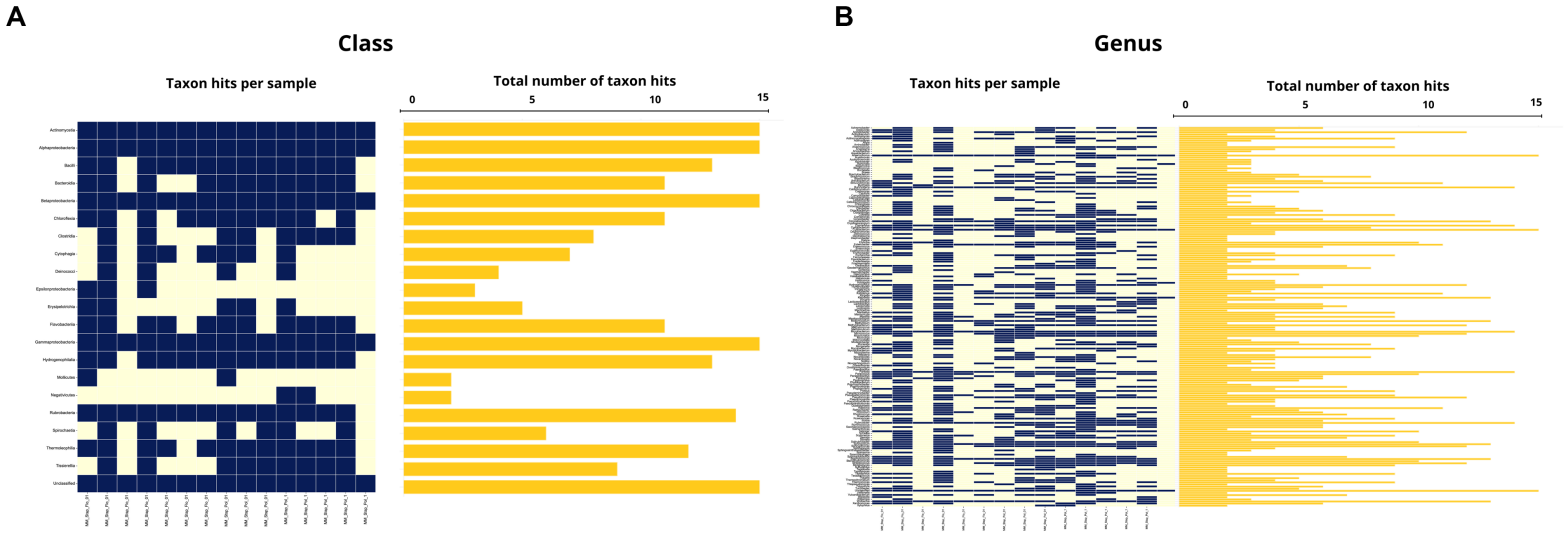
**(A)** Occurrence map of each taxon per sample at the class level found in the Macronyssidae mite samples. **(B)** Occurrence map of taxa at the genus level found in the Macronyssidae mite samples.

In macronyssids the most abundant bacterial classes were Gammaproteobacteria with an average abundance of 78.9% (1.2–99.2%) followed by Anctinomycetia and Alphaproteobacteria with average abundances of 15.8% (0.2–92%) and 3.22% (0.2–83.2%) respectively (Figures 7A, 8A). The most abundant bacterial taxa were *Arsenophonus,* with an average abundance of 77.3% (min 0.1–97.8%), followed by *Cutibacterium* with 13% (0.5–84.9%) and *Microbacterium* with 2.4% (0–28.8%) (Figures 7B, 8B).

The average abundance of unclassified taxa at class and genus levels, respectively, was 0.5% (0–5.6%) and 1.7% (0–16.6%) for Spinturnicidae and 0.7% (0.1–12.6%) and 2.4% (0.1–12.6%) for Macronyssidae.

#### 3.5.2. Bat flies

**Figure 9:**
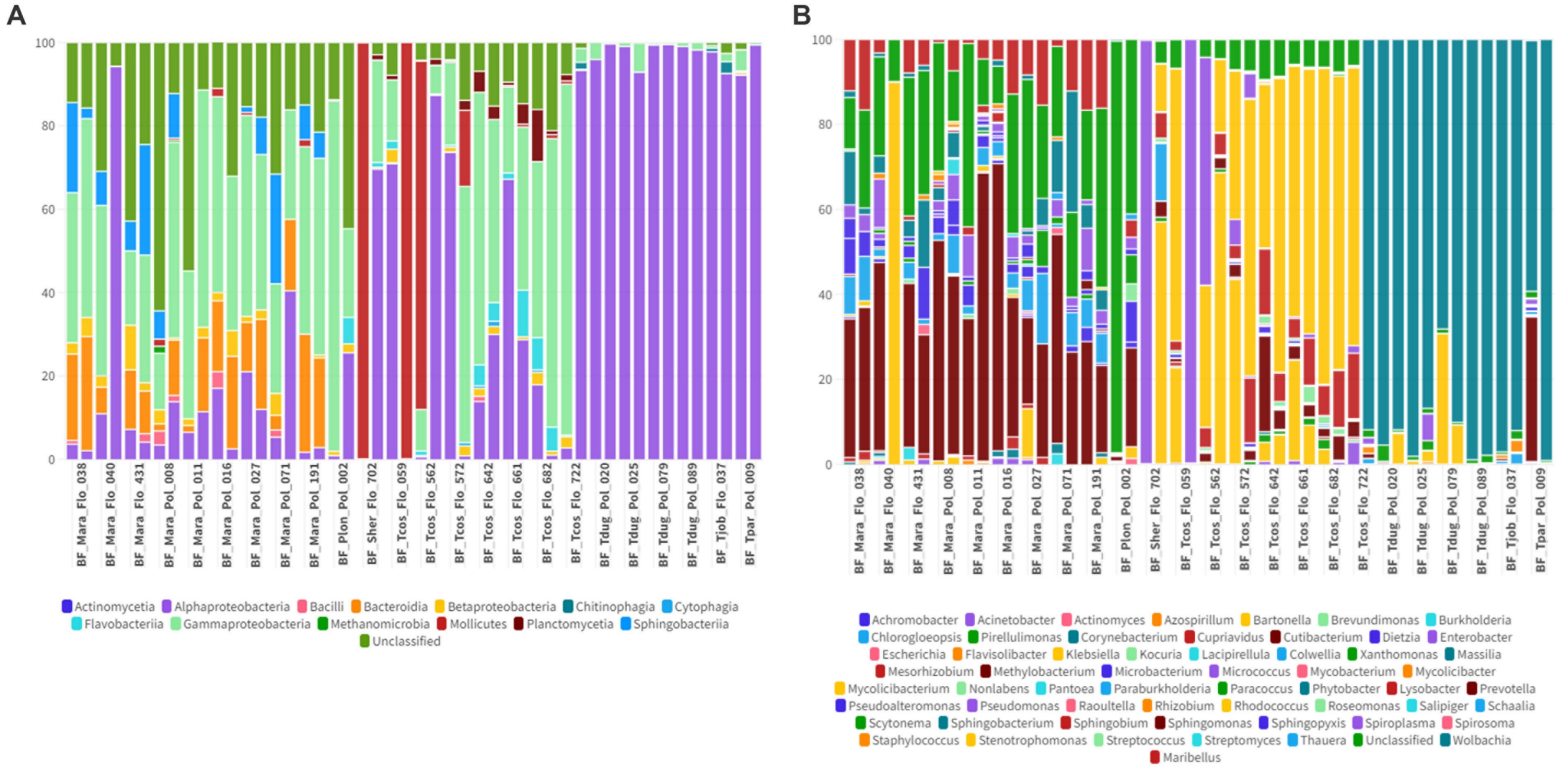
**(A)** Percentage of all taxa at the class level found in Streblidae flies collected from bats. **(B)** Percentage of all taxa at the genus level found in Streblidae flies collected from bats.

**Figure 10:**
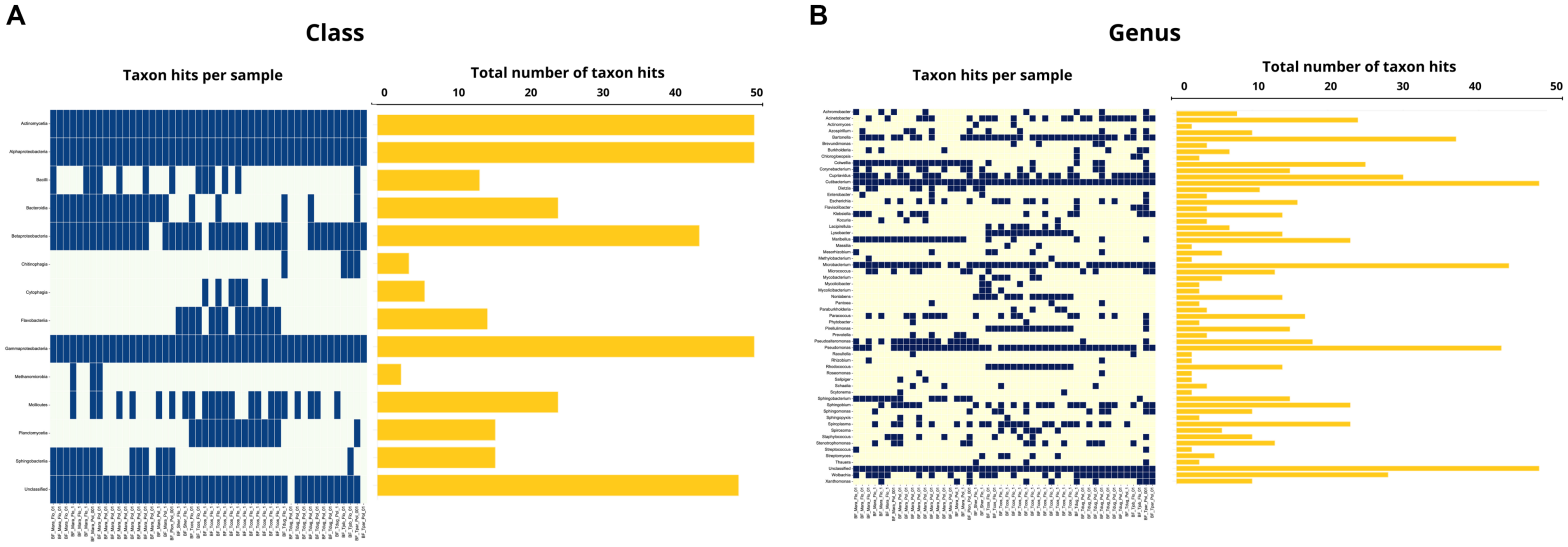
**(A)** Occurrence map of each taxon per sample at the class level found in the Streblidae fly samples. **(B)** Occurrence map of taxa at the genus level found in the Streblidae fly samples.

A total of 14 bacterial classes and 59 bacterial genera were detected in the Streblidae fly microbiomes. In the taxonomic classification at the class level, unclassified taxa showed an average abundance of 0.6% (0–28.7%). Among the most abundant taxa, Mollicutes showed an average abundance of 59.9% (0–99.9%) and Alphaproteobacteria and Gammaproteobacteria, present in all analyzed specimens, showed an average abundance of 36.6% (0.09–99.4%) and 1.2% (0.02–82%), respectively (Figures 9A, 10A). Regarding the taxonomic classification at the genus level, the most abundant were *Spiroplasma* (average abundance 59.9%; range 0–99.7%), *Wolbachia* (average abundance 26.7%; range 0–98.9%), *Bartonella* (average abundance 9.4%; range 0–89.9%), while we also observed a low relative abundance of unclassified taxa of 1.6% (0.01–96.8%) (Figures 9B, 10B).

## 4. Discussion

The microbiome composition of both oral (ranging from 13 to 86%) and rectal (ranging from 13 to 99%) swab samples showed that Gammaproteobacteria was the most abundant bacterial class, accounting for 42 and 70% of bacterial taxa, respectively. In addition to Gammaproteobacteria, other bacterial classes were also found in high relative abundance in both sort of samples, including (Firmicutes) Bacilli (26% in oral and 6% in rectal swabs), (Proteobacteria) Betaproteobacteria (8% in oral and 2.3% in rectal swabs) and (Actinomycetota) Actinomycetia (4% in oral and rectal swabs).

Lutz et al. (2019) also found a high relative abundance of Gammaproteobacteria in the oral and distal gut microbiome of insectivore and frugivore bats from Uganda and Kenya. Our findings were also similar to those found in previous studies performed in Mexico (Carrillo-Araujo et al., 2015), France (Hughes et al., 2018), southeastern Brazil (Cláudio et al., 2018), China (Xiao et al., 2019), and South Africa (Mehl et al., 2021), which have also found a high relative abundance of Proteobacteria in the gut microbiome of bats. Indeed, chiropterans have a higher proportion of Proteobacteria in their gut microbiome than other mammals, harboring a common core gut flora, irrespective of their geographic location or dietary habits. The reason for this difference is likely to be linked to the bat adaptation to flight and their rapid intestinal transit (Hughes et al., 2018; Nishida and Ochman, 2018; Jones et al., 2022).

Among the bacterial genera that were found in high abundance in rectal swab samples are *Enterobacter*, *Klebsiella*, *Escherichia*, and *Serratia* (Gammaproteobacteria: Enterobacteriaceae) as well as *Enterococcus* and *Streptococcus* (Bacilli: Streptococcaceae), which have also been detected in the rectal microbiome of *Myotis myotis* bats from Poland (Rózalska et al., 1998) and France (Hughes et al., 2018) and in the distal gut microbiome of *Rhinolophus monoceros* bats from India (Selvin et al., 2019). Although these bacteria play an essential role in the digestive system functioning, being present in the gut flora of most mammals, many may act as opportunistic pathogens to humans and animals (e.g., *Escherichia coli*, *Salmonella* sp., *Vibrio cholerae, Coxiella burnetii, Pseudomonas aeruginosa, Yersinia pestis*) (Garrity, 2007). Indeed, multidrug-resistant *Enterococcus faecalis* and *Escherichia coli* strains, which have been associated with human diseases, have already been found in frugivorous bats rectal and guano samples from Poland (Nowakiewicz et al., 2020, 2021). Similarly, novel strains of *Klebsiella africana* and *Klebsiella pneumoniae* were described in frugivorous *Pteropus poliocephalus* bats from Australia (McDougall et al., 2021), and septic-encephalitis causing *Streptococcus dysgalactiae* were detected in *Desmodus rotundus* from Brazil (Mioni et al., 2018). While the exact role of bats in transmitting pathogenic agents to humans is not yet fully understood, it is crucial that researchers conduct extensive studies on bat microbiomes and possible bat-bacteria associations. These studies will help predict the potential for bacterial antibiotic resistance and the transmission of pathogens to other animals and humans.

Similar to other mammals, bats have a diverse oral microbiome that is usually dominated by Firmicutes and Proteobacteria. Some studies have suggested that the oral microbiome of bats may be shaped by their diet, host phylogeny and environmental conditions (Carrillo-Araujo et al., 2015). Previous studies involving bats from Brazil (Cláudio et al., 2018) and South Africa (Dietrich et al., 2017) support our findings, indicating Proteobacteria as the most abundant phylum in oral samples. In contrast to our study, Presley et al. (2021) reported a predominance of Actinobacteria (30.6%) and Firmicutes (29.2%) in the oral microbiome of frugivorous and herbivorous bats from Puerto Rico, with only 0.4% of the sequences corresponding to Proteobacteria. Such a discrepancy could be attributed to environmental fluctuations, including factors such as diet, temperature, and humidity. These fluctuations can impact the diversity of microorganisms present in the bat oral microbiome (Klausenstock, 2016).

Out of the genera detected in oral swab samples, *Streptococcus*, *Neisseria*, *Actinomyces* and *Anaplasma* stand out among the most abundant taxa. Indeed, *Streptococcus* and *Neisseria* species are usually found in the oral cavity associated with dental and mucosal surfaces and saliva in mammals (Bennett et al., 2014; Abranches et al., 2018). Although previous studies have not reported the presence of *Anaplasma* species in the oral microbiome of bats, Gerbáčová et al. (2020) detected *Anaplasma* in the guano microbiome of *Myotis myotis* and *Rhinolophus hipposideros* bats from Slovakia. *Anaplasma phagocytophilum* DNA has already been detected in guano from horseshoe bat (*R. hipposideros*) maternity roosts in France (Afonso and Goydadin, 2018). In agreement with the abovementioned authors, a possible explanation for such a finding could be a persistent infection of bats with *Anaplasma*, with release of *Anaplasma* DNA in bat feces or, alternatively, caused by the consumption of arthropods containing the bacteria.

Although in our study both types of samples had comparable predominant taxa in common, it was possible to determine a higher bacterial diversity in rectal swab samples (21 classes and 189 genera) in comparison to oral swab samples (18 classes and 157 genera). There can be a few reasons why the rectal microbiome of bats is more diverse than the oral microbiome. One conceivable explanation is that the digestive system of bats is designed to extract as many nutrients as possible from their food, and this may lead to a higher bacterial richness in their gut (Nishida and Ochman, 2018). The anatomical structure of the digestive system may also play a role in shaping the microbial communities in different parts of the intestine (Hughes et al., 2018). Additionally, as already mentioned, the oral microbiome can be subjected to more environmental fluctuations, directly interfering with the bacterial richness. However, further investigation is required to fully understand the factors contributing to the differences in bacterial diversity between the oral and rectal microbiomes of bats.

The microbiome composition of the analyzed Spinturnicidae and Macronyssidae mites exhibited differences. Actinomycetia showed high relative abundance in both families (24.2% in spinturnicids and 15.8% in macronyssids), while Alphaproteobacteria dominated in spinturnicids (67.2%) and Gammaproteobacteria in macronissids (78.9%). Although there is limited research on the microbiome composition of these mites, especially those parasitizing bats, their microbial community composition is directly influenced by factors such as living environment, host bloodmeal source, and host species (Guo et al., 2020).

While *Wolbachia* (Alphaproteobacteria) was the most abundant genus found in Spinturnicidae (65.3%), *Arsenophonus* (Gammaproteobacteria) was found in high abundance in Macronyssidae (77.3%) mites. These two genera encompass symbiotic bacterial species found in many arthropods, such as flies, beetles, butterflies, ticks, bees, and wasps. They can establish either a commensal or mutualistic relationship with their hosts, providing protection against other infectious agents, such as parasitic nematodes, parasitoid wasps, fungi, and viruses (Kautz et al., 2013) However, in certain species, they can also act as pathogens, eliminating male progeny, inducing male feminization, and causing cytoplasmic incompatibility (Kautz et al., 2013). *Cutibacterium* (Actinomycetia) was found to be highly abundant in the microbiome composition of both mite families. The *Cutibacterium* genus, along with other closely related genera, such as *Acidipropionibacterium*, *Propionibacterium*, and *Pseudopropionibacterium*, are commensal microorganisms found on the skin of humans and animals and have been associated with the development of acne and various other skin conditions in humans (Patrick and Mcdowell, 2015; Mayslich et al., 2021). Although there are no reports on the presence of these bacteria in the skin microbiome composition of bats, Díaz-Sánchez et al. (2019) observed that *Cutibacterium* was the most prevalent genus in the microbiome of wild-caught *Ixodes ventalloi* ticks from Italy. The authors highlighted that *Cutibacterium* is commonly found in the microbiome of blood-feeding arthropods. These bacteria release volatile molecules from the sebaceous glands of their vertebrate hosts, which attract these ectoparasites and may be associated with their host-seeking behavior. It is worth noting that *Microbacterium* (Actinomycetia), which was also relatively abundant in the analyzed mite microbiome composition, has been reported in laboratory reagent contamination and can be overrepresented in microbiome analyses as a result (Salter et al., 2014).

Herein, the presence of *Bartonella* and *Anaplasma* was observed, in much lower abundances, in both Spinturnicidae and Macronyssidae families, whereas *Ehrlichia* was found only in Macronyssidae mites. The presence of *Bartonella* spp. in Spinturnicidae mites and associated bats have already been reported in China (Han et al., 2022). Some of the genotypes identified were shared between mites and bats, which could result from bloodsucking behavior. Furthermore, our research group has already detected by PCR followed by Sanger sequencing *Ehrlichia* sp. closely related to *Ehrlichia ruminantium* and *Ehrlichia minasensis* in the same Spinturnicidade and Macronyssidae mites used in the present work (Ikeda et al., 2021). Such difference in the results achieved by NGS and PCR followed by Sanger sequencing might have been due to different target genes used for each technique (*dsb* in PCR versus 16S rRNA in NGS). Although there is no current evidence supporting mites as competent to vector the abovementioned agents to bats, understanding the microbiome composition of these arthropods is crucial for advancing research on their microbial community compositions, vector competence and the epidemiology of arthropod-borne diseases.

A high abundance of Mollicutes sequences (59.9%), followed by Alpha (36.6%) and Gammaproteobacteria (1.2%), was found in the microbiome composition of the analyzed Streblidae bat flies. In contrast to our research findings, Speer et al. (2022) documented that Gammaproteobacteria (mostly *Arsenophonus*) constituted the most abundant class in the microbiome composition of Streblidae flies from southeastern Brazil. This observation aligns with the findings of Morse et al. (2012), who also reported Gammaproteobacteria (*Arsenophonus*) as the dominant class in flies from various regions including Puerto Rico, Panama, Peru, Costa Rica, Dominican Republic, Mexico, and the United States. The two most abundant genera observed in the present study were *Spiroplasma* (Mollicutes) and *Wolbachia* (Alphaproteobacteria), both of which are commonly found symbionts in various arthropod species (Cisak et al., 2015).

Following those genera, *Bartonella* was the third most abundant genus present in the microbiome composition. Out of the 48 flies analyzed, *Bartonella* sequences were detected in 37 (77%) specimens. Streblidae flies have already been identified as important hosts for many *Bartonella* genotypes and are possibly capable of transmitting the bacteria vertically (between themselves) or horizontally (between bats) (Morse et al., 2012; Do Amaral et al., 2018; Braga et al., 2020; Ikeda et al., 2020). Despite the high abundance of *Bartonella* sequences detected in Streblidae flies, we were unable to establish a connection between the microbiome of bat flies and bat oral and fecal swabs. Our findings revealed that only two bat swabs, one oral and one rectal, showed the presence of *Bartonella*. The observed lack of connection between infected flies and bats in terms of *Bartonella* sequences could potentially be attributed to the type of sample analyzed. It is worth noting that the prevalence of *Bartonella* in bats varies considerably, ranging from 7.3 to 54.4%, depending on the bat species, location and dietary niches and has already been documented in Africa, Americas, Asia and Europe (Stuckey et al., 2017). Furthermore, *Bartonella* species are known to be intraerythrocytic agents, with higher prevalence in blood-related samples. Since our study focused on oral and rectal swabs, which may not directly reflect the presence of *Bartonella* circulating in a bat bloodstream, it is possible that the detection of *Bartonella* in oral and rectal samples was limited. To obtain a more comprehensive understanding of the relationship between infected flies and bats, further investigations utilizing blood-related samples are much needed.

In summary, investigating the microbiome composition of bats and their ectoparasites provides valuable descriptive insights to guide future studies regarding the ecological dynamics of these organisms, as well as the potential for pathogen transmission and anti-microbial resistance. Future research should focus on understanding the interactions among bats, ectoparasites, and their associated microbiomes to better comprehend impacts on public health and wildlife conservation efforts.

## 5. Conclusion

The present study reports the oral and rectal microbiome composition of non-hematophagous bats and associated ectoparasites (Streblidae flies and Macronyssidae and Spinturnicidae mites) from Midwestern Brazil. In addition to the presence of symbionts, it was possible to observe the occurrence of bacterial classes and genera that include some species of animal and human medical importance. The microbiome composition of both oral and rectal bat swab samples showed that Gammaproteobacteria was the most abundant bacterial class. *Spiroplasma, Wolbachia* and *Bartonella* represented the most abundant genera in Streblidae flies. While *Wolbachia* (Alphaproteobacteria) was the most abundant genus found in Spinturnicidae, *Arsenophonus* (Gammaproteobacteria) was found in high abundance in Macronyssidae mites. In addition to characterizing the microbiome of each sample at the class and genus taxonomic levels, our results reveal the potential presence of medically significant bacteria able to infect both animals and humans. This includes those identified in oral (*Streptococcus* and *Anaplasma*) and rectal swabs (*Enterobacter*, *Klebsiella*, *Escherichia*, *Enterococcus*, *Streptococcus*), Macronyssidae (*Anaplasma*, *Bartonella*, *Ehrlichia*) and Spinturnicidae (*Anaplasma*, *Bartonella*) mites as well as Streblidae flies (*Spiroplasma*, *Bartonella*). Besides expanding the knowledge of the bacterial microbiome of non-hematophagous bats and Streblidae flies from Brazil, the present work describes the bacterial community of bat-associated Macronyssidae and Spinturnicidae mites.

## Data availability statement

The datasets presented in this study can be found in online repositories. The names of the repository/repositories and accession number(s) can be found at this link: https://dataview.ncbi.nlm.nih.gov/object/PRJNA1011525?reviewer=i6m1ps7ghm3i13r476ov2270q0 repository, accession number PRJNA1011525.

## Ethics statement

The animal study was approved by ICMBio – SISBIO number 57450-1 and Ethics Committee on the Use of Animals (CEUA – FCAV/UNESP), registered under number 010050. The study was conducted in accordance with the local legislation and institutional requirements.

## Author contributions

MA: Conceptualization, Funding acquisition, Investigation, Project administration, Resources, Writing – original draft, Writing – review & editing. PI: Conceptualization, Investigation, Methodology, Writing – review & editing. DL: Investigation, Visualization, Writing – original draft, Writing – review & editing. RA: Investigation, Methodology, Software, Visualization, Writing – original draft, Writing – review & editing. LC: Data curation, Formal analysis, Investigation, Methodology, Software, Writing – review & editing. DP: Conceptualization, Data curation, Formal analysis, Investigation, Methodology, Software, Validation, Writing – review & editing. JT: Methodology, Writing – review & editing. VM: Methodology, Writing – review & editing. GR: Writing – review & editing, Methodology. RC: Writing – review & editing, Methodology. EL: Methodology, Writing – review & editing. CO: Methodology, Writing – review & editing. HH: Methodology, Writing – review & editing. DB-B: Methodology, Writing – review & editing. RM: Investigation, Writing – review & editing. KB-L: Writing – review & editing, Methodology, Funding acquisition, Resources. CD: Methodology, Resources, Validation, Writing – review & editing. JD: Formal analysis, Funding acquisition, Investigation, Methodology, Project administration, Resources, Supervision, Writing – review & editing.

## Author disclaimer

The opinions expressed herein are those of the author(s) and are not necessarily representative of those of the Uniformed Services University of the Health Sciences (USUHS), the Department of Defense (DOD); or, the United States Army, Department of the Navy, Air Force, nor the U.S. Government. Several authors are federal employees of the United States government. This work was prepared as part of their official duties. Title 17 U.S.C. §105 provides that ‘Copyright protection under this title is not available for any work of the United States Government.’ Title 17 U.S.C. §101 defines a U.S. Government work as a work prepared by a military service member or employee of the U.S. Government as part of that person’s official duties.
