# Supplementary material for: Characterization of the bacterial microbiome of non-hematophagous bats and associated ectoparasites from Brazil
Source: Front Microbiol. 2023 Oct 19;14:1261156. doi: 10.3389/fmicb.2023.1261156 (PMC10620512; doi:10.3389/fmicb.2023.1261156)
Supplement: Supplementary file 1 [file Table_1.DOCX]

**Supplementary Material**

**Table SM1.** Mites and their associated bat species.

| Family | Mite Species | Associated host | Number of specimens collected |
| --- | --- | --- | --- |
| Macronyssidae | *Steatonyssus* sp. | *Eptesicus furinalis* | 6 |
|  |  | *Eumops perotis* | 3 |
|  |  | *Phyllostomus discolor* | 3 |
|  |  | *Artibeus lituratus* | 1 |
|  |  | *Artibeus planirostris* | 1 |
|  |  | *Molossus molossus* | 1 |
| Spinturnicidae | *Periglischrus iheringi* | *Artibeus lituratus* | 15 |
|  |  | *Platyrrhinus lineatus* | 8 |
|  |  | *Artibeus planirostris* | 6 |
|  | *Periglischrus* sp. | *Phyllostomus discolor* | 4 |
|  |  | *Eptesicus furinalis* | 1 |
|  | *Periglischrus torrealbai* | *Phyllostomus discolor* | 4 |
|  | *Periglischrus acutisternus* | *Phyllostomus discolor* | 4 |

**Table SM2.** Streblidae flies and their associated bat species.

| Fly Species | Associated host | Number of specimens collected |
| --- | --- | --- |
| *Megistopoda aranae* | *Artibeus planirostris* | 16 |
|  | *Artibeus lituratus* | 2 |
| *Trichobius costalimai* | *Phyllostomus discolor* | 14 |
| *Trichobius dugesii* complex | *Platyrrhinus lineatus* | 6 |
|  | *Artibeus lituratus* | 1 |
|  | *Carollia perspicilliata* | 1 |
|  | *Eptesicus furinalis* | 1 |
| *Trichobius joblingi* | *Carollia perspicilliata* | 2 |
| *Trichobius parasiticus* complex | *Glossophaga soricina* | 1 |
|  | *Platyrrhinus lineatus* | 1 |
| *Strebla hertigi* | *Phyllostomus discolor* | 2 |

**Table SM3.** Metagenome Read and Assembly Statistics.

| **Metagenome** | **Bat Swabs (BS)** | **Bat Flies (BF)** | **Macronyssidae Mites**  **(MM)** | **Spinturnicidae Mites**  **(SM)** |
| --- | --- | --- | --- | --- |
| Raw reads  (pairs) | 262,664,416 | 248,329,927 | 74,899,048 | 219,444,431 |
| After QC reads  (pairs) | 250,661,629 | 248,285,140 | 70,944,122 | 219,423,506 |
| No. Contigs | 4,252,226 | 1,721,435 | 522,339 | 6,943,159 |
| No. Contigs  (> 1 Kbp) | 1,112,465 | 331,870 | 186,878 | 1,051,134 |
| No. Contigs  (> 10 Kbp) | 39,450 | 10,413 | 8,837 | 1,209 |
| Largest Contig | 346,543 | 763,712 | 272,723 | 38,249 |
| N50 | 3,798 | 3,536 | 4,693 | 1,688 |
| L50 | 212,426 | 46,575 | 36,503 | 337,042 |
| Mapped (%) | 95.67 | 98.08 | 89.93 | 85.70 |
| Avg. coverage depth | 20 | 72 | 23 | 25 |
